# Carbohydrate Intake Practices and Determinants of Food Choices During Training in Recreational, Amateur, and Professional Endurance Athletes: A Survey Analysis

**DOI:** 10.3389/fnut.2022.862396

**Published:** 2022-03-11

**Authors:** Corinne Reinhard, Stuart D. R. Galloway

**Affiliations:** Physiology, Exercise and Nutrition Research Group, Faculty of Health Sciences and Sport, University of Stirling, Stirling, United Kingdom

**Keywords:** sport nutrition, sports drinks, gels, energy bars, food, carbohydrate, training, endurance athletes

## Abstract

Carbohydrate (CHO) intake during exercise can optimize endurance performance. However, there is limited information regarding fueling practices of endurance athletes during training. Accordingly, an anonymous German-language online survey was circulated examining the determinants of CHO choices, and intake practices among runners, triathletes, and cyclists during training. Survey questions included predefined answers, and a Likert scale with response of CHO food choice intakes from 1 = never to 5 = always. 1,081 endurance participants (58.0% male, 68.6% aged 18–39 years) of varying competitive levels were included in the analysis. Overall, most participants consumed a combination of commercial sport nutrition products and everyday foods (67.4%, *n* = 729) with their primary reason that food-first was preferred, but in some exercise scenarios, commercial sport nutrition products were deemed more convenient (61.3%, *n* = 447). Participants consuming commercial sport nutrition products only (19.3%, *n* = 209) most often valued their ease of intake during exercise (85.2%, *n* = 178). Among those consuming everyday foods only (13.2%, *n* = 143), the most common reason was the perceived importance of eating wholesome foods/natural ingredients (84.6%, *n* = 121). Between the most frequently consumed CHO sources during training at low-to-moderate intensities (*n* = 1032), sports drinks (mean ± SD; 2.56 ± 1.33) were consumed significantly more often than bananas (2.27 ± 1.14, *p* < 0.001), with no significant difference in intake frequency between bananas and traditional muesli/fruit/energy bars (2.25 ± 1.14, *p* = 0.616). Whereas during high intensities (*n* = 1,077), sports drinks (3.31 ± 1.51) were significantly more often consumed than gels (2.79 ± 1.37), and gels significantly more often than energy bars (2.43 ± 1.28), all commercial sport nutrition products (all, *p* < 0.001). Overall, 95.1% (*n* = 1028) of all participants consumed CHO during training at all exercise intensities, with males (*n* = 602; 2.35 ± 0.70) consuming significantly more often commercial sport nutrition products than females (*n* = 424; 2.14 ± 0.79, *p* < 0.001); females consumed significantly more often everyday foods than males (1.66 ± 0.47 vs. 1.54 ± 0.42, *p* < 0.001). Most participants used mixed CHO forms during low-to-moderate (87.9%), and high exercise intensities (94.7%). 67.6% (*n* = 731) of all participants reported guiding their CHO intake rates during training by gut feeling. These large-scale survey findings suggest a preference of endurance participants’ CHO intake during training in liquid form independent of exercise intensities and offer novel insights into CHO intake practices to guide sports nutrition strategies and education.

## Introduction

Carbohydrate (CHO) ingestion during exercise has an ergogenic effect on endurance performance (1–3). Furthermore, there is support that adequate CHO intake during prolonged and intense endurance exercise beneficially influences immune function (4). In contrast, reduced endogenous and/or exogenous CHO availability can promote endurance training-induced adaptations (5). In practice, athletes’ individual reason(s) for CHO intake and their applied CHO feeding schedule during endurance training sessions may not only be influenced by factors such as training status and sports nutrition knowledge, but also depend on different training goals such as aiming for improvement of exercise performance, training of the gut, or practice of race fueling strategies.

To optimize exercise fueling strategies, the amount, type, and form of CHO intake needs to be considered and individualized according to the athlete’s specific training situation and goal, their gut comfort, and their individual preferences. Contemporary guidelines for CHO intake rates during exercise by the American College of Sports Medicine (ACSM) to promote optimal performance are based on the indication of grams of CHO per hour of exercise: small amounts of CHO, including mouth rinse, are recommended during sustained high exercise intensities over 45–75 min, 30–60 g CHO/h for endurance exercise durations of 1–2.5 h, and up to 90 g CHO/h for prolonged endurance exercise of >2.5–3 h (6). Recent findings have suggested that some athletes might benefit from CHO intake rates of up to 120 g/h during exercise (7). It has been recognized, that depending on absolute exercise intensity, recommendations for CHO intake rates might need to be adjusted downward if absolute intensity is low (e.g., slower athletes) (8). Optimal CHO intake rates for beneficial outcomes might not only be exercise intensity specific (9), but also depend on CHO type. For target CHO intakes >60–70 g/h, only multiple transportable CHOs (i.e., mixtures of glucogenic and fructogenic sources) should be used to allow for high oxidation rates (1, 8) and to improve gastrointestinal comfort (10), while single-source fast-oxidized CHO (e.g., glucose) can also be considered for lower (<60 g/h) CHO intake rates/h. However, to the best of our knowledge, data on how endurance athletes guide their CHO intake rates during training, and whether the current recommendation of using grams of CHO/h calculations has been adopted in training, is lacking.

Studies have shown similar CHO oxidation efficiencies for CHO-containing liquids, semi-solids, or easily digestible (low fat, low fiber, and low protein) solid *foods* with a glucose + fructose ratio of 2:1 (11, 12). In a review by Cermak and van Loon (13), it was concluded that the CHO supplement form, whether in a liquid, semi-solid, or solid state, did not alter the ergogenic effects of CHO intake during prolonged exercise. While available information in athletes’ usage of CHO forms during endurance events suggest that most athletes use mixed CHO forms (14–18), published data on the choice of CHO forms during training situations among triathletes, runners, or cyclists are currently lacking. CHO intake during exercise is achievable through suitable everyday food options or commercial sports foods – food products specifically formulated for and marketed to athletes. For a long time, such sport nutrition products were primarily meant for and used by elite athletes. Today, their usage is widespread at all levels, including among amateur and recreational athletes, along with non-exercisers. CHO-rich sport nutrition products are commonly sold in easy portable formats such as gels, chews, bars, and sachets, primarily designed to meet the demands for convenience of endurance and team sport athletes and are often claimed to be easy digestible to support gut comfort. In general, the disadvantages of sports foods include their potentially high cost and the risk of ingesting banned substances in sports, sometimes present as contaminants (19). Athletes are advised to follow a ‘food first philosophy‘ (i.e., everyday food versus supplements and sports foods), as was recently highlighted by the International Association of Athletics Federations consensus statement (20). Data collected during endurance events have shown that most athletes rely on a mix of commercial sport nutrition products and everyday foods for CHO intake (18, 21, 22). Very limited published data are available for training situations: in a survey by Heikura et al. (23), 90% of 50 elite middle- and long-distance runners/race walkers who reported undertaking training sessions with CHO ingestion used sport nutrition products solely. Furthermore, in their previous pilot study (24), none of the elite middle- and long-distance endurance athletes (*n* = 14) who consumed CHO during workouts reported using CHO-rich whole foods. These two studies showed that athletes had a clear preference for commercial sport nutrition products compared to everyday foods when undertaking training sessions with CHO. Data during training from a larger sample size including athletes of varying training backgrounds and different types of endurance sport are lacking. Furthermore, the rationale given by endurance athletes for choosing commercial CHO-rich sport nutrition products and/or CHO-containing everyday foods to fuel their training sessions has not yet been examined.

Gaining a better understanding of endurance athletes’ CHO fueling practices and their reasons for food choices during training could help nutritionists and coaches optimize sports nutrition strategies and education for athletes. It could also provide important insights into the food industry for product development. Therefore, the primary aim of this study was to examine the prevalence and determinants of the use of solely CHO-rich commercial sport nutrition products, solely everyday foods, or a combination thereof during training sessions undertaken with CHO in a large sample of triathletes, cyclists, and runners with varying training backgrounds and competitive levels. Furthermore, we wished to identify the specific CHO food choices and the choice in CHO forms of these athletes during training at low-to-moderate, and high exercise intensities. A secondary aim was to identify the practices applied by endurance athletes to guide their CHO intake rates during exercise. A final aim was to assess whether differences in CHO practices related to sex, competitive level and types of endurance sport exist.

## Materials and Methods

### Survey

This study was conducted as a cross-sectional anonymous German-language online survey using SurveyMonkey software (Advantage version). It was completed between May 2021 and June 2021, over a total of 4 weeks. Participants were recruited using a snowball sampling procedure, which involved posting the invitation to participate in the research on social media (Facebook, Instagram, Twitter, and LinkedIn) and the circulation of the survey link via e-mail and WhatsApp to key direct contacts in Germany, Austria, and Switzerland, including sports dietitians and sports coaches, athletes, and organizers of endurance events. The survey was reviewed for face validity by two sports dietitians and one sports scientist, piloted among five athletes with different endurance training and nutrition backgrounds, and updated based on the feedback received. Skip logic function was used to allow respondents to navigate only to questions relevant to them (according to their answers) to avoid confusion and to improve their participation experience. Participants were asked to answer between 12 and 14 questions in total. The Ethics Committee of the University of Stirling approved the study, which conformed to the Declaration of Helsinki.

Prior to the beginning of the survey, details about the study were provided, and all participants completed an informed consent. The themes explored in the online survey included the following: (A) General background (demographics, training status, and training history). The options for participants to report their competitive level included recreational (train but at most take part in regional races), amateur (participation in national or international races), and professional. Based on the very recently published framework to classify research participants performance caliber (25), our recreational level covers Tier 1 (Recreational) and 2 (Trained/Developmental), while the amateur level covers Tier 3 (Highly Trained/National Level) and 4 (Elite/International Level) and our professional level is classified as Tier 4 and 5 (World Class); (B) Choice of CHO food category (solely everyday foods, solely commercial sport nutrition products, or a combination of commercial sport nutrition products and everyday foods) and the corresponding determinants. A set of predefined answers for participants’ reasons why they chose what they chose were presented, including an open-ended answer option for individual responses. The predefined answers were presented in a randomized order and developed by the authors; they were influenced by the factors (convenience, functionality, gut comfort, price, social influences, familiarity, natural concerns, doping-risk, and sensory appeal), that are perceived or had been shown as relevant to food choice in the general and athlete populations in previous studies (26–30); (C) CHO choices and intake frequencies during training, dependent on training intensity. For exercise intensity classification, unless otherwise controlled, perceived exertion was accordingly rated as light to somewhat hard (for low-to-moderate exercise intensities) and hard to extremely hard (for high exercise intensities). A 5-point Likert scale (never, rarely, sometimes, often, and always) for predefined CHO sources was provided, along with an open-ended answer opportunity; (D) Participant’s applied practices regarding guidance of CHO intake rates; predefined answers and an open-ended answer option were provided. The original survey is provided in the Supplementary Material of this manuscript.

### Participants

Active German-speaking triathletes, cyclists, and runners of a recreational up to a professional level were invited to participate. Eligibility criteria included participants: (a) ≥18 years old, (b) at least occasionally consuming CHO during training sessions, and (c) providing consent to participate.

### Statistical Analysis

Only data from completed surveys were considered and further cleaned by excluding participants who provided clearly contrasting responses or individual responses for “other” that did not satisfy the overall requirement of the question.

Descriptive data and further statistical analyses were conducted using Jamovi and SPSS software [version 1.6.23; SPSS version 28.0]. Data are presented as percentage (%), number (*n*), or means ± standard deviation (SD) of responses. Additionally, data are presented as percentiles (25th, 50th, and 75th) where appropriate. Statistical significance was set at *p* < 0.05, and 95% confidence intervals (CI) were also presented. To investigate associations of categorical data and CHO food categories (only commercial sport nutrition products, only everyday foods, or a combination thereof), and participants’ applied CHO intake rate practices, Pearson Chi^2^ tests for independence were used, along with Cramer’s V effect size statistic. Cramer’s V coefficient strength associations are interpreted as negligible (<0.10), weak (0.10 to <0.20), moderate (0.20 to <0.40), relative strong (0.40 to <0.60), strong (0.60 to <0.80), and very strong (0.80–1.00) (32). Where significant effects were found, *z*-tests were performed as *post hoc* tests with Bonferroni correction. For determinants of CHO food categories, percentages (%) were calculated as per the *n* of the corresponding CHO food category. Participants could select multiple answers for this question; therefore, the sum of % of responses exceeded 100%. The 5-point Likert scale for CHO food sources was ranked as 1 = never, 2 = rarely, 3 = sometimes, 4 = often, and 5 = always. To test for differences between the three most often used CHO food sources during training at low-to-moderate, and at high exercise intensities, Friedman tests were used and where significant effects were found, pairwise comparisons were analyzed using Durbin-Conover tests. For differences in intake frequencies of solid, semi-solid, and liquid foods and of commercial sport nutrition products and everyday foods between low-to-moderate and high exercise intensities, Wilcoxon test was applied, and *R* effect size statistic was interpreted as small (=0.10), medium (=0.30), and large (=0.50) (33). To examine sex-differences in intake frequencies of CHO food choices and overall intake frequencies of commercial sport nutrition products and everyday foods, Mann–Whitney *U* tests were applied, and R effect sizes were also presented. Differences between types of sport, and competitive level of participants in overall intake frequency of commercial sport nutrition products, and everyday foods were assessed by Kruskal–Wallis tests and where significant effects were found, Dwass-Steel-Critchlow-Fligner pairwise *post hoc* comparisons were used.

Two diverse participants and one participant who declined to reveal sex, were omitted from sex-based analyses. Individual answers that were filled in for “other,” i.e., open-ended answer opportunities, were grouped into common subgroups for reporting but were not included in analyses.

## Results

### Participants

**FIGURE 1:**
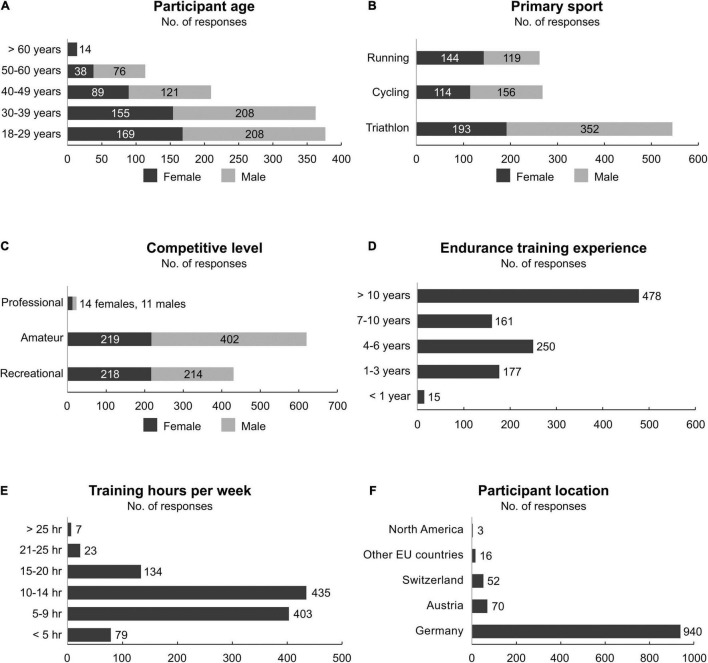
**(A)** Age, **(B)** primary sport, **(C)** competitive level, **(D)** endurance training experience in years, **(E)** typical weekly training volume, and **(F)** participant location. *N* = 1,081, except **(A–C)** where two participants (amateur triathlete aged 18–29 years, and recreational cyclist aged 50–60 years) are diverse, and one participant (amateur triathlete aged 18–29 years) declined to give sex. For **(F)**, participants grouped by European (EU) countries were from nine different countries. The North America participants were from United States and Canada.

A total of 1,081 participants (41.7% female, 58.0% male, 0.2% diverse, 0.1% declined to give sex; aged 18 to >60 years with 68.6% of participants being 18–39 years old, Figure 1A), were included in the final analysis. The sports represented were triathlon (50.6%), cycling (25.1%) and running (24.1%, Figure 1B). Of all participants, 40.1% classified themselves as recreational, 57.6% as amateur, and 2.3% as professional participants (Figure 1C). Of the recreational participants, 52.0% (*n* = 225) reported general fitness and health, 44.8% (*n* = 194) reported performance improvement, and 3.2% (*n* = 14) reported other reasons (including pleasure, combination of general fitness and health and performance improvement, and weight reduction) as primary motive for training. Only 1.4% of all participants had less than 1 year of experience in endurance training, 16.4% had between 1 and 3 years, 23.1% between 4 and 6 years, 14.9% between 7 and 10 years, and 44.2% more than 10 years (Figure 1D). The typical weekly volume the participants trained was <5 h (7.3%), 5–9 h (37.3%), 10–14 h (40.2%), 15–20 h (12.4%), 21–25 h (2.1%), and >25 h (0.6%, Figure 1E). A total of 98.2% of the participants resided in Germany, Austria, and Switzerland (Figure 1F).

### Prevalence and Determinants of Carbohydrate Food Choices

Overall, most respondents reported using a combination of commercial sport nutrition products and everyday foods (67.4%, *n* = 729) followed by solely commercial sport nutrition products (19.3%, *n* = 209) and by solely everyday foods (13.2%, *n* = 143) for CHO intake during training sessions. Significant subgroup associations were found between sex [χ^2^(2) = 31.731, *p* < 0.001, 95% CI (0.11, 0.23)], type of sport [χ^2^(4) = 52.276, *p* < 0.001, 95% CI (0.12, 0.20)], and competitive level [χ^2^(4) = 51.630, *p* < 0.001, 95% CI (0.12, 0.21)] of participants, with weak associations based on Cramer’s V (all <0.20). Males (70.5%) were more likely to use a combination of commercial sport nutrition products and everyday foods than females (63.2%; *p* = 0.012), while intake of only everyday foods was significantly more common among females (20.0%) than among males (8.3%; *p* < 0.001). Triathletes (6.8%) were less likely to use solely everyday foods than runners (23.2%; *p* < 0.001) or cyclists (16.6%; *p* < 0.001). CHO intake through commercial sport nutrition products only was more popular among triathletes (23.4%) than among cyclists (13.7%; *p* = 0.003). Triathletes (69.8%) were also more likely to use a combination of commercial sport nutrition products and everyday foods than runners (60.1%; *p* = 0.017). Recreational participants (21.7%) were more likely to use solely everyday foods than amateurs (7.9%; *p* < 0.001) or professional participants (0.0%; *p* < 0.05). However, the use of solely commercial sport nutrition products was more popular among amateurs (22.2%) than among recreational participants (14.5%; *p* = 0.006).

**FIGURE 2:**
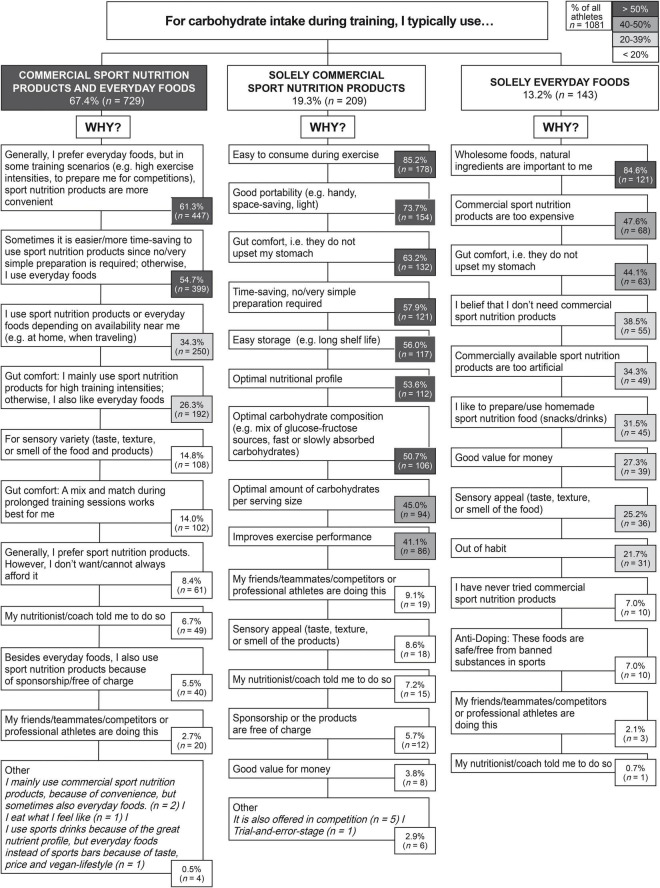
Reasons for using a combination of commercial sport nutrition products and everyday foods, solely commercial sport nutrition products, and solely everyday foods for carbohydrate intake during training sessions. Percentages reflect the % of participants who chose a specific answer in relation to all participants who were presented with a given question.

Among participants who reported using a combination of commercial sport nutrition products and everyday foods they most often reported that “food-first” is preferred, but in some exercise scenarios, commercial sport nutrition products were deemed more convenient (*n* = 447, 61.3%; Figure 2). In those who solely used commercial sport nutrition products for CHO intake during training, the most common reasons were related to their convenience, i.e., ease to consume during exercise (*n* = 178, 85.2%) and good portability (*n* = 154, 73.7%; Figure 2). Wholesome foods/natural ingredients were of primary importance for endurance participants consuming only everyday foods (*n* = 121, 84.6%) (Figure 2). Of all participants (*n* = 1,081), 15.0% (*n* = 162) considered the sensory appeal of food as a determinant for their food choice. In total, 11.9% (*n* = 129) of all participants reported the factor price as a reason for never or not exclusively using commercial sport nutrition products during training. Furthermore, only 3.9% (*n* = 42) of all participants cited social influences by friends, teammates, competitors, or professional athletes as a reason for their CHO food category choice during training sessions, and only 6.0% (*n* = 65) of all participants chose their CHO food category because their nutritionist or coach told them to do so (Figure 2).

### Intake Frequencies of Carbohydrate Foods

Of all participants (*n* = 1,081), 4.5% (*n* = 49) did not consume CHO during low-to-moderate exercise intensities, and four recreational participants (0.4%) did not consume CHO during training of high intensities but did during low-to-moderate intensities. CHO food choices and their intake frequencies among endurance participants during training at low-to-moderate exercise intensities (*n* = 1,032) are shown in Table 1, and those during high exercise intensities (*n* = 1,077) are shown in Table 2. From the Likert scale responses, the most frequently consumed CHO sources during training at low-to-moderate intensities included sports drinks (2.56 ± 1.33), bananas (2.27 ± 1.14), and traditional muesli/fruit/energy bars (2.25 ± 1.14, Table 1). Significant effects were found between these three most often consumed food sources [χ^2^(2) = 30.165, *p* < 0.001]. CHO intake in the liquid form of sports drinks was significantly more often than in solid form of bananas (*p* < 0.001), or traditional muesli/fruit/energy bars (*p* < 0.001), with no significant difference between the two solid foods (*p* = 0.616). During training at high exercise intensities (*n* = 1,077), sports drinks (3.31 ± 1.51), gels (2.79 ± 1.37), and energy/CHO bars (2.43 ± 1.28), all commercial sport nutrition products, were preferred (Table 2), with significant effects between these food sources [χ^2^(2) = 317.144, *p* < 0.001]. CHO was significantly more often consumed in liquid form of sports drinks than in semi-solid (gels), or solid (energy bars) form, and significantly more often in the semi-solid than solid form (all, *p* < 0.001). Differences in intake frequencies of CHO food choices between males and females are shown for training at low-to-moderate exercise intensities in Table 3, and at high exercise intensities in Table 4. In total, 95.1% (*n* = 1,028) of all participants reported to consume CHO in training sessions at both exercise intensities, with everyday foods being consumed significantly more often at low-to-moderate exercise intensities (1.60 ± 0.46) than at high exercise intensities (1.58 ± 0.50, *p* = 0.024, *r* = 0.095, small effect). In contrast, intake of commercial sport nutrition products was significantly more often during high exercise intensities (2.54 ± 0.89) than during low-to-moderate exercise intensities (1.98 ± 0.72, *p* < 0.001, *r* = 0.882, large effect). Males (*n* = 602; 2.35 ± 0.70) reported using commercial sport nutrition products significantly more often than females (*n* = 424; 2.14 ± 0.79, *p* < 0.001, *r* = 0.165, small effect). In contrast, everyday foods were consumed significantly more often by females (1.66 ± 0.47) during training than by males (1.54 ± 0.42, *p* < 0.001, *r* = 0.144, small effect). Further significant subgroup differences were also found between the type of sport and competitive level of participants: Commercial sport nutrition products were significantly less often consumed by runners (*n* = 237; 1.90 ± 0.70) than cyclists (*n* = 264; 2.38 ± 0.84, *p* < 0.001) or triathletes (*n* = 527; 2.37 ± 0.65, *p* < 0.001), with no significant difference in intake frequency between triathletes and cyclists (*p* = 0.802). Everyday foods were significantly more popular in cyclists (1.74 ± 0.46) than in runners (1.61 ± 0.47, *p* < 0.001) or in triathletes (1.51 ± 0.41, *p* < 0.001), and significantly more often consumed in runners than in triathletes (*p* = 0.021). With regard to the competitive level of participants, recreational participants (*n* = 410; 2.09 ± 0.78) used commercial sport nutrition products less often than amateur participants (*n* = 595; 2.38 ± 0.70, *p* < 0.001) or professional participants (*n* = 23; 2.51 ± 0.67, *p* = 0.028), with no significant difference in intake frequency between amateur and professional participants (*p* = 0.710). However, everyday foods were more often consumed by recreational participants (1.66 ± 0.44) than amateur participants (1.54 ± 0.44, *p* < 0.001). No significant differences in intake frequency of everyday foods were found between recreational and professional participants (1.56 ± 0.43, *p* = 0.606), or between professional and amateur participants (*p* = 0.920).

**TABLE 1:** Intake frequencies of carbohydrate foods in endurance participants (*n* = 1,032) during training at low-to-moderate intensities.

CHO foods and forms	25th centile	50th centile	75th centile	Mean (SD)	1. Never	2. Rarely (max. 10% of training sessions undertaken with CHO)	3. Sometimes (up to 50% of training sessions undertaken with CHO)	4. Often (>50% of training sessions undertaken with CHO)	5. Always (all training sessions undertaken with CHO)
**Liquid form**									
Sports drinks*	1	2	4	2.56 ± 1.33	27.9%	26.4%	18.2%	17.3%	10.2%
Homemade sports drinks	1	1	2	1.83 ± 1.15	57.1%	18.4%	11.2%	10.6%	2.7%
Sweetened beverages, saftschorle	1	1	2	1.40 ± 0.74	71.9%	19.5%	5.6%	2.7%	0.3%
Coke, energy drinks	1	1	1	1.34 ± 0.66	75.2%	17.4%	5.9%	1.4%	0.1%
Other: Coconut water						0.1%			
**Semi-solid form**									
Gels*	1	2	2	1.83 ± 0.95	46.5%	31.4%	15.3%	6.1%	0.7%
Fruit puree pouches	1	1	1	1.23 ± 0.62	84.5%	9.8%	3.8%	1.7%	0.2%
**Solid form**									
Energy/carbohydrate bars*	1	2	3	2.14 ± 1.10	36.3%	28.7%	21.3%	11.6%	2.0%
Muesli/fruit, or other energy bars	1	2	3	2.25 ± 1.14	33.4%	27.4%	22.5%	14.1%	2.6%
Homemade sweet snacks (e.g., rice cakes)	1	2	3	2.19 ± 1.12	35.5%	27.2%	21.9%	13.4%	2.0%
Sweet baked goods (e.g., muffins, cake)	1	1	2	1.46 ± 0.74	67.2%	21.9%	8.9%	1.8%	0.1%
Bread/rolls/lye pastry, pure, or with spread	1	1	2	1.55 ± 0.83	64.1%	21.0%	11.2%	3.7%	n/a
Sandwiches	1	1	1	1.27 ± 0.61	80.2%	13.8%	4.7%	1.3%	n/a
Banana	1	2	3	2.27 ± 1.14	33.8%	25.4%	23.4%	15.1%	2.2%
Dried fruits (e.g., raisins, dates)	1	1	2	1.56 ± 0.89	65.3%	19.7%	9.4%	5.3%	0.3%
Sports nutrition confectionery (e.g., chews/gums)*	1	1	2	1.39 ± 0.71	71.5%	20.0%	6.4%	2.0%	0.1%
Confectionery (e.g., jelly babies, chocolate)	1	1	2	1.40 ± 0.69	70.2%	22.1%	5.7%	1.9%	0.1%
Other: Potatoes						0.1%	0.1%		

Percentages reflect the % of participants who consumed carbohydrates in training during low-to-moderate intensities (n = 1,032). CHO, carbohydrate. *Denotes commercially available sport nutrition products.

**TABLE 2:** Intake frequencies of carbohydrate foods in endurance participants (*n* = 1,077) during training at high intensities.

CHO foods and forms	25th centile	50th centile	75th centile	Mean (SD)	1. Never	2. Rarely (max. 10% of training sessions undertaken with CHO)	3. Sometimes (up to 50% of training sessions undertaken with CHO)	4. Often (>50% of training sessions undertaken with CHO)	5. Always (all training sessions undertaken with CHO)
**Liquid form**									
Sports drinks*	2	4	5	3.31 ± 1.51	21.8%	9.4%	13.3%	27.3%	28.2%
Homemade sports drinks	1	1	3	1.93 ± 1.31	58.6%	13.1%	11.0%	10.9%	6.4%
Sweetened beverages, saftschorle	1	1	2	1.46 ± 0.86	71.8%	16.0%	7.6%	3.5%	1.1%
Coke, energy drinks	1	1	2	1.57 ± 0.94	67.2%	16.2%	9.7%	6.3%	0.5%
**Semi-solid form**									
Gels*	1	3	4	2.79 ± 1.37	26.3%	16.0%	22.4%	23.4%	12.0%
Fruit puree pouches	1	1	1	1.22 ± 0.65	87.3%	6.8%	3.4%	2.0%	0.5%
Other: Homemade gels								0.1%	
**Solid form**									
Energy/carbohydrate bars*	1	2	3	2.43 ± 1.28	33.9%	19.3%	22.8%	17.9%	6.0%
Muesli/fruit, or other energy bars	1	2	3	2.13 ± 1.23	44.7%	19.4%	18.8%	13.1%	4.1%
Homemade sweet snacks (e.g., rice cakes)	1	1	2	1.53 ± 0.89	67.4%	17.7%	9.7%	4.5%	0.7%
Sweet baked goods (e.g., muffins, cake)	1	1	1	1.36 ± 0.73	76.5%	14.4%	6.5%	2.2%	0.4%
Bread/rolls/lye pastry, pure, or with spread	1	1	1	1.36 ± 0.75	77.2%	13.2%	6.3%	3.2%	0.1%
Sandwiches	1	1	1	1.20 ± 0.57	86.7%	8.3%	3.6%	1.3%	0.1%
Banana	1	2	3	2.13 ± 1.24	44.8%	20.2%	15.3%	16.2%	3.4%
Dried fruits	1	1	2	1.48 ± 0.91	72.9%	13.0%	7.9%	5.5%	0.7%
Sports nutrition confectionery (e.g., chews/gums)*	1	1	1	1.55 ± 0.96	69.5%	13.9%	10.1%	5.0%	1.4%
Confectionery (e.g., jelly babies, chocolate)	1	1	2	1.42 ± 0.81	73.6%	15.4%	7.0%	3.4%	0.6%
Other: Potatoes								0.1%	

Percentages reflect the % of participants who consumed carbohydrates in training during high intensities (n = 1,077). CHO, carbohydrate. *Denotes commercially available sport nutrition products.

**TABLE 3:** Sex-based differences in intake frequencies of carbohydrate foods during training at low-to-moderate exercise intensities.

CHO foods and forms	Female (*n* = 426)		Male (*n* = 604)		
	50th centile	Mean (SD)	50th centile	Mean (SD)	*p-*value, *r*
**Liquid form**					
Sports drinks*	2	2.30 ± 1.32	3	2.74 ± 1.30	***p* < 0.001, *r* = 0.200**
Homemade sports drinks	1	1.85 ± 1.16	1	1.82 ± 1.14	*p* = 0.626, *r* = 0.016
Sweetened beverages, saftschorle	1	1.40 ± 0.76	1	1.40 ± 0.73	*p* = 0.850, *r* = 0.005
Coke, energy drinks	1	1.22 ± 0.50	1	1.42 ± 0.74	***p* < 0.001, *r* = 0.123**
**Semi-solid form**					
Gels*	1	1.60 ± 0.89	2	1.99 ± 0.95	***p* < 0.001, *r* = 0.251**
Fruit puree pouches	1	1.31 ± 0.72	1	1.18 ± 0.52	***p* = 0.002, *r* = 0.070**
**Solid form**					
Energy/carbohydrate bars*	2	2.10 ± 1.13	2	2.18 ± 1.07	*p* = 0.112, *r* = 0.056
Muesli/fruit, or other energy bars	2	2.40 ± 1.18	2	2.15 ± 1.10	***p* < 0.001, *r* = 0.116**
Homemade sweet snacks (e.g., rice cakes)	2	2.34 ± 1.16	2	2.09 ± 1.08	***p* < 0.001, *r* = 0.116**
Sweet baked goods (e.g., muffins, cake)	1	1.41 ± 0.70	1	1.49 ± 0.77	*p* = 0.093, *r* = 0.051
Bread/rolls/lye pastry, pure, or with spread	1	1.62 ± 0.88	1	1.50 ± 0.80	***p* = 0.034, *r* = 0.066**
Sandwiches	1	1.32 ± 0.67	1	1.24 ± 0.56	***p* = 0.037, *r* = 0.053**
Banana	2	2.34 ± 1.19	2	2.22 ± 1.11	*p* = 0.127, *r* = 0.054
Dried fruits	1	1.78 ± 1.01	1	1.39 ± 0.75	***p* < 0.001, *r* = 0.212**
Sports nutrition confectionery (e.g., chews/gums)*	1	1.36 ± 0.70	1	1.41 ± 0.71	*p* = 0.131, *r* = 0.044
Confectionery (e.g., jelly babies, chocolate)	1	1.37 ± 0.66	1	1.41 ± 0.71	*p* = 0.264, *r* = 0.033

CHO, carbohydrate. *Denotes commercially available sport nutrition products. Significant differences (p < 0.05) are given in bold. R effect sizes interpreted as = 0.10 (small effect), = 0.30 (medium effect), and = 0.50 (large effect).

**TABLE 4:** Sex-based differences in intake frequencies of carbohydrate foods during training at high exercise intensities.

CHO foods and forms	Female (*n* = 449)		Male (*n* = 625)		
	50th centile	Mean (SD)	50th centile	Mean (SD)	*p*-value, *r*
**Liquid form**					
Sports drinks*	3	3.08 ± 1.56	4	3.48 ± 1.44	***p* < 0.001, *r* = 0.143**
Homemade sports drinks	1	1.97 ± 1.31	1	1.90 ± 1.30	*p* = 0.306, *r* = 0.033
Sweetened beverages, saftschorle	1	1.51 ± 0.88	1	1.43 ± 0.85	*p* = 0.060, *r* = 0.053
Coke, energy drinks	1	1.44 ± 0.83	1	1.66 ± 1.00	***p* < 0.001, *r* = 0.112**
**Semi-solid form**					
Gels*	2	2.49 ± 1.42	3	3.01 ± 1.29	***p* < 0.001, *r* = 0.211**
Fruit puree pouches	1	1.29 ± 0.75	1	1.16 ± 0.56	***p* < 0.001, *r* = 0.074**
**Solid form**					
Energy/carbohydrate bars*	2	2.47 ± 1.34	2	2.41 ± 1.24	*p* = 0.590, *r* = 0.019
Muesli/fruit, or other energy bars	2	2.32 ± 1.30	2	1.99 ± 1.16	***p* < 0.001, *r* = 0.138**
Homemade sweet snacks (e.g., rice cakes)	1	1.70 ± 0.99	1	1.41 ± 0.80	***p* < 0.001, *r* = 0.157**
Sweet baked goods (e.g., muffins, cake)	1	1.37 ± 0.74	1	1.35 ± 0.73	*p* = 0.507, *r* = 0.018
Bread/rolls/lye pastry, pure or with spread	1	1.49 ± 0.86	1	1.27 ± 0.65	***p* < 0.001, *r* = 0.128**
Sandwiches	1	1.26 ± 0.65	1	1.16 ± 0.50	***p* = 0.003, *r* = 0.063**
Banana	2	2.26 ± 1.28	2	2.04 ± 1.21	***p* = 0.007, *r* = 0.091**
Dried fruits	1	1.72 ± 1.06	1	1.31 ± 0.74	***p* < 0.001, *r* = 0.207**
Sports nutrition confectionery (e.g., chews/gums)*	1	1.60 ± 1.03	1	1.51 ± 0.91	*p* = 0.293, *r* = 0.031
Confectionery (e.g., jelly babies, chocolate)	1	1.42 ± 0.80	1	1.41 ± 0.81	*p* = 0.978, *r* = 0.001

CHO, carbohydrate. *Denotes commercially available sport nutrition products. Significant differences (p < 0.05) are given in bold. R effect sizes interpreted as = 0.10 (small effect), = 0.30 (medium effect), and = 0.50 (large effect).

### Form of Carbohydrate Intake

Carbohydrate was consumed in liquid, semi-solid, and solid forms (Tables 1, 2). During training at low-to-moderate exercise intensities (*n* = 1,032), most participants consumed CHO in mixed forms of different combinations (87.9%, *n* = 907): of these participants, 59.8% (*n* = 542) had a mixed usage of all three CHO forms, i.e., liquid, semi-solid, and solid state. Only 12.1% of participants used exclusively either solid (*n* = 75), liquid (*n* = 41) or semi-solid (*n* = 9) CHO forms. Similarly, during training at high exercise intensities (*n* = 1,077), most participants consumed CHO in mixed forms of different combinations (94.7%, *n* = 1,020): of these participants, 70.2% (*n* = 716) used all three CHO forms. Only 5.3% of participants reported using either solely solid (*n* = 29), solely liquid (*n* = 22), or solely semi-solid (*n* = 6) CHO forms during high exercise intensities. Interestingly, among the three CHO intake forms, semi-solids (gels, fruit puree pouches) had the highest percentage of never being used by participants during both training intensities, that is, 40.3% of participants did not report any intake during low-to-moderate intensities, and 23.3% of participants did not report any intake during high exercise intensities. Participants, who consumed CHO during training at both exercise intensities (*n* = 1,028), consumed liquids (2.54 ± 1.07 vs. 2.09 ± 0.88, *p* < 0.001, *r* = 0.780, large effect), and semi-solid foods (2.23 ± 1.05 vs. 1.65 ± 0.75, *p* < 0.001, *r* = 0.887, large effect) significantly more often at high exercise intensities than at low-to-moderate exercise intensities. However, there was no significant difference in the intake frequency of solid foods (high intensities: 1.85 ± 0.62; low-to-moderate intensities: 1.82 ± 0.53) between exercise intensities (*p* = 0.125; *r* = 0.060, small effect).

### Carbohydrate Intake Rate Practices During Exercise

**FIGURE 3:**
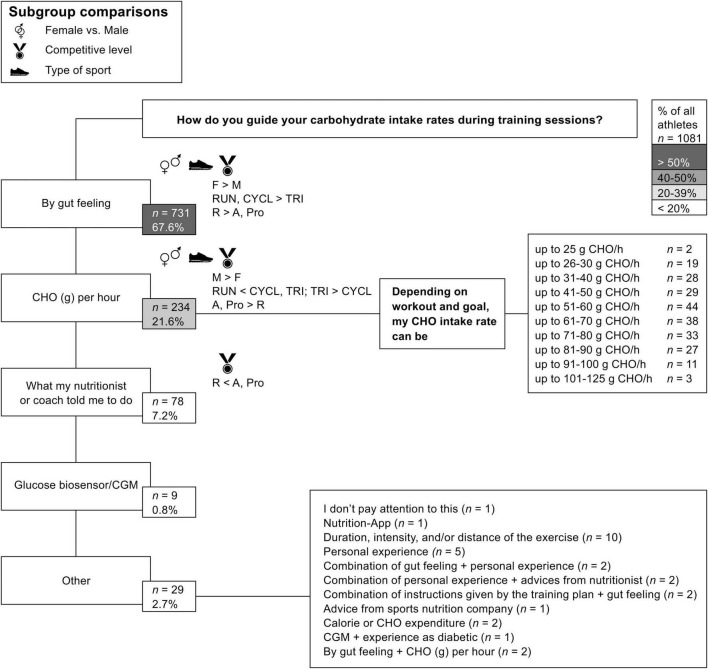
Applied carbohydrate (CHO) intake rate practices during training sessions among endurance participants (*n* = 1,081). CGM, continuous glucose monitoring. Significant differences between subgroups (*p* < 0.05) are depicted with symbols and a brief description of the direction of the differences. F, female; M, male; R, recreational; A, amateur; Pro, professional; RUN, running; CYCL, cycling; TRI, triathlon.

Overall, more than half of all participants (67.6%, *n* = 731) reported guiding their CHO intake rates during training sessions undertaken with CHO by gut feeling (Figure 3). In contrast, only 21.6% (*n* = 234) of all participants monitored their CHO requirements using grams of CHO/h calculations, with approximately half of them having intake rates of up to 60 g CHO/h (52%, *n* = 122) and of up to more than 60 g of CHO/h (48%, *n* = 112) during training (Figure 3). A small number of all participants (10.7%, *n* = 116) reported relying on the advice of their nutritionists or coaches, using glucose biosensors/continuous glucose monitoring, or guiding their CHO intake rates based on other aspects (Figure 3). Significant subgroup associations were found between sex [χ^2^(3) = 26.338, *p* < 0.001, Cramer’s V = 0.158, 95% CI (0.11, 0.22), weak association], type of sport [χ^2^(6) = 40.280, *p* < 0.001, Cramer’s V = 0.138, 95% CI (0.11, 0.18), weak association], and competitive level [χ^2^(6) = 86.101, *p* < 0.001, Cramer’s V = 0.202, 95% CI (0.17, 0.25), moderate association] (Figure 3). Females (74.7%) were more likely to guide their CHO intake rates by gut feeling than males (62.5%; *p* < 0.001), and the approach of using grams of CHO/h calculations was significantly more often reported in males (27.1%) than in females (14.2%; *p* < 0.001). Among triathletes (59.6%), guidance of CHO intake rates by gut feeling was less popular than among cyclists (72.7%; *p* = 0.004) or runners (79.1%; *p* < 0.001). The guidance of CHO requirements by using grams of CHO/h calculations was more likely in triathletes (27.8%) than in cyclists (19.9%; *p* = 0.023), and it was less likely in runners (10.6%) than in cyclists (*p* = 0.012) and triathletes (*p* < 0.001). Regarding the trends across competitive levels, significantly more recreational participants (82.9%) reported using their gut feeling to guide their CHO intake rates than amateurs (58.1%; *p* < 0.001) or professional participants (40.0%, *p* < 0.001). Further, recreational participants (10.2%) were less likely to calculate grams of CHO per hour than amateur (28.9%; *p* < 0.001) or professional participants (40.0%, *p* < 0.001) to guide their CHO requirements. In addition, only 3.9% of recreational participants reported guiding their CHO intake rates based on advice from their nutritionist or coach, compared to 9.0% of amateurs (*p* = 0.004) or 20% of professional participants (*p* = 0.001).

## Discussion

This is the first large-scale survey (*n* = 1,081) describing the prevalence and determinants of CHO choices and intake practices during training in endurance participants. The habits of CHO intake practices during training and reasons for their choices related to solely commercial sport nutrition products, solely everyday foods, or a combination of these food categories are characterized among German-speaking triathletes, runners, and cyclists across a range of ages, endurance training backgrounds, and competitive levels. There are multiple main findings in this study: (1) Most participants reported using a combination of commercial sport nutrition products and everyday foods for their CHO intake during endurance training. (2) Commercial sport nutrition products were more frequently used by males than females and during training at high intensities than low-to-moderate intensities, while the converse was true regarding everyday foods intake. (3) Participants’ primary determinant for consuming commercial sport nutrition products during training was convenience, with a general preference in consuming everyday foods in those who used commercial sport nutrition products in combination with everyday foods. Wholesome foods and natural ingredients are of utmost importance in participants using only everyday foods during training. Social influences by peers and advice from their nutritionists or coaches were less of a reason in all participants for their food choices during training. (4) Most participants used a mix of solid, semi-solid, and liquid forms for CHO intake during training at any exercise intensity. (5) Sports drinks (commercial sport nutrition product) are the most often consumed CHO source during any training intensity, although more frequently consumed during high exercise intensities. This was followed by bananas, and traditional muesli/fruit/energy bars during low-to-moderate exercise intensities, and by a more frequent intake of gels than energy/CHO bars (both commercial sport nutrition products) during high exercise intensities. (6) Participants used a greater variety of CHO foods dominated by a more frequent use of liquid and semi-solid CHO forms, more often during training at high exercise intensities than at low-to-moderate intensities. (7) Only few endurance participants use calculations of grams of CHO/h of exercise to guide their CHO intake rates during training. (8) Nearly all outcomes relating to CHO intake practices differed by sex, competitive level, and type of sport.

The results of this survey show that most endurance participants use a combination of commercial sport nutrition products and everyday foods for CHO intake during training, independent of competitive level. The current findings contrast with previous surveys of elite middle- and long-distance athletes reporting that nearly all consume solely commercial sport nutrition products during workouts (23, 24), but might be related to the athletes’ competitive status being world-class level in these two previous studies. However, these previous observations that almost all professional athletes never rely on the sole use of everyday foods were confirmed in our sub-sample of professional participants as the sole use of everyday foods for CHO intake was never reported. Overall, 86.8%, (*n* = 938) of all participants in our survey reported to consume CHO-rich commercial sport nutrition products, either in combination with everyday foods or solely during endurance training. These observations are in line with other studies, showing that, in general, there is widespread use of sports foods in athletic populations (34). Maughan et al. (35) reported that surveys generally suggest a higher usage of dietary supplements in male athletes than female athletes, and an increased usage with level of training/performance. We also found that in participants undertaking training sessions with CHO intake at any training intensity, commercial sport nutrition products were more often consumed by males, amateurs, and professional participants during training than by females and recreational participants. In contrast, females and recreational participants consumed everyday foods more often than males and amateur participants. However, our analysis included more male amateur participants (*n* = 402) than female amateur participants (*n* = 218), which might have influenced these findings.

To our knowledge, this is the first time, determinants for food choices, intake frequencies of CHO sources, the usage of CHO forms, and applied practices to guide CHO intake rates during training of endurance participants have been examined. Our survey findings have shown that convenience characteristics are of primary motive in participants for using commercial sport nutrition products only during training. A preference for “food-first,” but the use of commercial sport nutrition products in specific training situations when everyday foods are impractical was the most cited reason of participants when commercial sport nutrition products were used in combination with everyday foods. In support, a central philosophy of “food-first” including the sound usage of sports foods, is recommended in the recent International Olympic Committee (IOC) consensus statement on dietary supplements and the high-performance athlete (35). However, in the present study it remains unclear why the food-first approach is preferred in these participants, i.e., whether the reasons are related to factors such as health or sustainability, or to minimize the anti-doping risk (36). Wholesome foods and natural ingredients of foods were of utmost importance in participants for considering only everyday foods to cover CHO needs in training. This is not surprising, as a growing interest in “clean” eating and concerns about artificial ingredients in the athletic and general population can be observed in the media, as well as a clean labeling trend within the (sports) food industry (37, 38). Our survey findings also show that many participants consider gut comfort when making CHO food choices during training. Previous experience with gastrointestinal issues, a common complaint in endurance athletes (16, 39), may influence their CHO food choices. Interestingly, social influences by peers or advice from their nutritionists/coaches were not common reasons given by participants for their decision to use commercial sport nutrition products and/or everyday foods for CHO intake during training. However, these findings are not dissimilar to others that have surveyed endurance athletes on source of nutritional advice. For example, Doering et al. (40) reported that most amateur triathletes rely on their own knowledge, while in the study by McLeman et al. (41), most amateur runners used the Internet for sports nutrition information. However, if the participants’ rationales for their choices in our survey are influenced by other information sources such as media (e.g., the internet) or by confidence in making independent food decisions, because of previous personal experience, remains unclear. Thurecht and Pelly (30) reported that sensory appeal was a key factor for food choice in athletes at the 2017 Universiade and 2018 Commonwealth Games. In our study, only 15% of all participants considered the sensory appeal of food as a determinant for their CHO food choices during training. However, this discrepancy may be related to the timing of food intake. In support, Birkenhead and Slater (27) concluded in their study that athletes’ food selection is influenced by taste, but importance likely varies with eating occasion. Taken together, our preliminary findings on the underlying reasons of participants’ food decisions for CHO intake during training provides valuable information for product development of the food industry, and for nutritionists/coaches to develop sport nutrition education and strategies.

Most participants in the present study reported using a mix of different CHO forms over single CHO forms during training. Similar observations were made during endurance events (14–18). The recently published Europe Sports Drink Market Report (42) indicated a growth in the segment of sports drinks during the next years. Fueling and hydration are key elements of nutrition during exercise. Benefits of CHO-containing sports drinks that might be attractive to athletes include the simultaneous intake of fluid, CHO, electrolytes, and optionally with additional ingredients such as caffeine and the ease of intake (e.g., no chewing needed) during exercise. Furthermore, there are many options on the sports nutrition market, such as mix-with-water tablets or powders, including a variety of different formulations (related to CHO composition, and ingredients) and a wide range of flavors to fit individuals’ needs. Indeed, sports drinks in the form of a commercial sport nutrition product were the most often consumed CHO source at any training intensity in our survey, although more frequently consumed during training at high exercise intensities. These findings also suggest a preference for endurance participants’ CHO intake during training in liquid form. However, further research is warranted to verify the preference in the form of CHO intake during endurance training at different exercise intensities, durations, and environmental conditions. Furthermore, participants reported use of a greater variety of CHO foods more often at high exercise intensities than at low-to-moderate exercise intensities, but these were dominated by a more frequent use of liquid and semi-solid CHO forms during high exercise intensities. However, it remains unclear whether these outcomes result from a higher preference in mix-and-match strategies of different CHO foods, or because greater amounts of CHO are consumed during training at high exercise intensities. Thus, it seems that semi-solid and liquid foods are considered more convenient than solid foods during high exercise intensities. The results of the Likert scale responses also show that the majority of participants did not use the same CHO source during all training sessions (i.e., “always”) for fueling at low-to-moderate exercise intensities. In contrast, this was more common during training at high exercise intensities. These findings suggest that endurance participants may choose their CHO sources more flexibly and pragmatically during training at lower exercise intensities than higher exercise intensities. However, further research is required. As a practical recommendation, athletes should select their CHO food form(s) and sources during exercise based on personal preferences, tolerance (gut comfort), and the practical opportunities provided in an event or training session to obtain and consume CHO, along with sufficient fluid intake. Mix-and-match strategies of CHO forms and sources can be implemented as a possible nutrition strategy to achieve individual CHO targets.

In our study, only ∼20% of participants guided their CHO intake rates during training in accordance with the approach of contemporary sport nutrition guidelines, recommending the use of grams of CHO/h of exercise calculations to promote optimal performance (6). More than half of all participants reported guiding their CHO intake rates during training by gut feeling. It is worth mentioning that for just over half of all recreational participants the intention for training was general fitness and health, implying that focusing on an optimal CHO intake strategy to improve endurance performance might not be as important for this group of athletes as for others. Taken together, these results suggest: (A) endurance participants’ need greater awareness, including effective education about evidence-based approaches for CHO intake rate strategies to support optimal training performance; and (B) further research is required to investigate whether endurance participants amount and type of CHO intake during training might be in line with current guidelines, despite not using the approach of grams of CHO/h calculations.

Despite our large sample size, we acknowledge some limitations of this study largely related to non-controlled responses, including the description of self-reported sport nutrition practices in our online survey. Self-reporting of dietary intake can be biased by social desirability (43). Furthermore, data collection happened during the COVID-19-pandemic. These factors might have affected how respondents considered certain items, independent of their true habits/behavior, and the ensured participant anonymity of our online survey we had provided. It is therefore possible that survey responses concerned with the determinants that participants perceive as relevant to their choice of using commercial sport nutrition products and/or everyday foods or reported intake frequencies of CHO foods during training do not necessarily reflect actual dietary behavior. It is also possible that the respondents who chose to participate in our survey may not represent all endurance participants. Although we had prevented multiple votes per participant (the survey could be taken only once from the same device), online surveys generally pose the risk of a self-selection bias (44). Other limitations include the small sample size of professional participants and the fact that most participants surveyed were residents in Germany, Austria and Switzerland. Thus, the findings of the present study might not translate to other countries and cultures or all endurance participants.

### Conclusion

To our knowledge, this is the first large-scale survey examining carbohydrate intake practices and determinants of food choices during training of runners, triathletes, and cyclists, across a range of ages, endurance training backgrounds, and competitive levels. We conclude that:

• Most endurance participants use a combination of commercial sport nutrition products and everyday foods for their fueling requirements during training. Intake of commercial sport nutrition products during training is more common in males and at high exercise intensities. In contrast, the use of everyday foods for CHO intake during training is more common in females and during low-to-moderate exercise intensities.
• Convenience characteristics are the primary reasons for using commercial sport nutrition products, with a general preference for everyday foods when taking both. Further, wholesome foods/natural ingredients are a key determinant in endurance participants for solely using everyday foods during training. In contrast, social influences by friends, teammates, competitors, or professional athletes, and advice from a nutritionist or coach appear not to be relevant determinants for food choices during training in all endurance participants.
• Most endurance participants use a mix of liquid, semi-solid, and solid CHO forms during training at all exercise intensities. Sports drinks as a commercial sport nutrition product are the most frequently consumed CHO source during training at all exercise intensities, although more frequently consumed at high exercise intensities. A greater variety of CHO foods, dominated by a more frequent intake of liquid and semi-solid forms is used more often during higher exercise intensities. Furthermore, our findings suggest that endurance participants may choose their CHO sources more flexibly and pragmatically during training at lower exercise intensities than higher exercise intensities.
• Most endurance participants do not guide their CHO intake rates during training according to current sport nutrition guidelines.

Nearly all objectives measured relating to CHO intake practices varied by sex, competitive level, and type of sport. These large-scale survey findings offer novel insights into CHO intake practices and determinants of food choices in endurance participants during training for nutritionists/coaches to guide sport nutrition strategies and education, and for sport nutrition manufacturers to design products and communication.

## Data Availability Statement

The raw data supporting the conclusions of this article will be made available by the authors, without undue reservation.

## Ethics Statement

The studies involving human participants were reviewed and approved by Ethics Committee of the University of Stirling. The patients/participants provided their written informed consent to participate in this study.

## Author Contributions

CR and SG designed the survey and undertook the manuscript preparation. CR collected and analyzed the data. Both authors contributed to the article and approved the submitted version.

## Conflict of Interest

The authors declare that the research was conducted in the absence of any commercial or financial relationships that could be construed as a potential conflict of interest.

## Publisher’s Note

All claims expressed in this article are solely those of the authors and do not necessarily represent those of their affiliated organizations, or those of the publisher, the editors and the reviewers. Any product that may be evaluated in this article, or claim that may be made by its manufacturer, is not guaranteed or endorsed by the publisher.
