# Supplementary material for: Carbohydrate Intake Practices and Determinants of Food Choices During Training in Recreational, Amateur, and Professional Endurance Athletes: A Survey Analysis
Source: Front Nutr. 2022 Mar 11;9:862396. doi: 10.3389/fnut.2022.862396 (PMC8963786; doi:10.3389/fnut.2022.862396)
Supplement: Supplementary file 1 [file Data_Sheet_1.PDF]

UNIVERSITY of STIRLING SPORT

Online-Umfrage zum Kohlenhydratverzehr von Ausdauersportlern während des Trainings

Lieber Ausdauersportler, liebe Ausdauersportlerin,

bitte unterstütze uns dabei, die Häufigkeit und Motive der Kohlenhydratwahl von Ausdauersportlern (Freizeit-, Amateur- bis Profiniveau) im Training zu erforschen.

Du bist herzlich eingeladen, an dieser kurzen anonymen Online-Umfrage teilzunehmen, die ca. 7-10 Minuten dauert.

Teilnahmevoraussetzungen:

- Du bist mindestens 18 Jahre alt
- Du bist Freizeit-, Amateur- oder Profisportler (m/w/d) im Bereich Triathlon, Laufen oder Radfahren
- Du nimmst gelegentlich (oder immer) kohlenhydrathaltige Snacks und/oder Getränke (z.B. Banane, Gels, Brötchen, Sportgetränke, Cola…) in Trainingseinheiten zu Dir

Bevor Du Dich entscheidest, an dieser Studie teilzunehmen, lies bitte die nachfolgenden Informationen sorgfältig durch.

Zweck dieser Studie
Kohlenhydrate zählen zu den wichtigsten Energielieferanten für Ausdauersportler. Unseres Wissens nach, hat jedoch bisher keine Studie die spezifische Kohlenhydratwahl von Ausdauersportlern im Training untersucht. Die Zielsetzung dieses Projektes ist es daher zu erforschen, welche Kohlenhydratlieferanten (Snacks/Getränke) Ausdauersportler in Trainingssituationen konsumieren und aus welchen Gründen diese Entscheidungen getroffen werden. Die Ergebnisse dieser Studie werden zur Sporternährungsforschung beitragen und neue Erkenntnisse liefern, wie Ausdauersportler mit unterschiedlichem Background während dem Training „Energie tanken".

Unsere Bitte: Nimm Dir 7-10 Minuten Zeit
Wir möchten Dich bitten, eine kurze und anonyme Online-Multiple-Choice-Umfrage auszufüllen. Bei Trainingseinheiten, in denen Du Kohlenhydrate konsumierst, möchten wir wissen „was" Du zu Dir nimmst und „warum". Mit Deiner Unterstützung trägst Du zur Sporternährungsforschung bei.
Die Forschungsarbeit wird in einem wissenschaftlichen Journal publiziert und kann in jeglicher Art von Berichten verwendet sowie auf nationalen/internationalen wissenschaftlichen Konferenzen präsentiert werden. Eine Infografik über die Zusammenfassung der Studienergebnisse wird über soziale Medien verbreitet - unter anderem über die Accounts von Stuart Galloway und Corinne Reinhard.

Weitere Informationen
Dieses Forschungsprojekt läuft in Kooperation mit der University of Stirling, Schottland und wurde von der Ethikkommission genehmigt. Deine Teilnahme an dieser Studie ist freiwillig. Diese Online-Umfrage läuft komplett anonymisiert ab. Die Daten dieser Studie werden in anonymisierter Form erhoben, ausgewertet und mindestens 10 Jahre gespeichert. Bei Fragen kannst Du gerne eine E-Mail an den Projektleiter senden: corinnereinhard@icloud.com.
Du hast das Recht, bei der britischen Datenschutzbehörde ICO (https://ico.org.uk/concerns/) eine Beschwerde gegen die Universität in Bezug auf Datenschutz einzureichen. Bei Fragen zum Datenschutz, wende Dich bitte zunächst an data.protection@stir.ac.uk.

Herzlichen Dank für Deine Unterstützung,
Corinne Reinhard & Dr. Stuart Galloway

***Dies ist kein von der Industrie finanziertes Forschungsprojekt. Das Forschungsteam erklärt, dass kein Interessenkonflikt besteht.**

UNIVERSITY of STIRLING SPORT

Einwilligungserklärung

Bevor wir mit der Umfrage beginnen, möchten wir Dich auf Deine Rechte als Teilnehmer in diesem Forschungsprojekt hinweisen. Bitte lies Dir die folgenden Informationen sorgfältig durch:

Hiermit erkläre ich folgendes:

- Ich bin mindestens 18 Jahre alt.
- Ich wurde über die Art des Forschungsprojektes, dessen Zweck und Umfang ausreichend informiert. Zusätzliche Informationen zu dieser Studie kann ich anfordern, indem ich eine E-Mail an corinnereinhard@icloud.com sende.
- Ich bin darüber aufgeklärt worden, dass die Teilnahme an der Studie freiwillig ist und ich jederzeit ohne Angabe von Gründen von der Studie zurücktreten kann.
- Mir ist bekannt, dass diese Studie von der Ethikkommission der Universität Stirling, Schottland, genehmigt wurde und dass diese Studie gemäß der Deklaration von Helsinki durchgeführt wird.
- Ich erkläre mich damit einverstanden, dass die erhobenen, anonymen Daten dieser Studie für mindestens zehn Jahre gespeichert werden. Die anonymisierten Daten werden zur Auswertung und für jede Art von Dokumentationen, Berichten, Veröffentlichungen oder wissenschaftlicher Literatur im Rahmen dieser Studie verwendet.

☐ Ich bin einverstanden und möchte an der Umfrage teilnehmen

UNIVERSITY of STIRLING SPORT

Wie alt bist Du?

- ◯ 18-29 Jahre
- ◯ 30-39 Jahre
- ◯ 40-49 Jahre
- ◯ 50-60 Jahre
- ◯ > 60 Jahre

Ich bin...

- ◯ Weiblich
- ◯ Männlich
- ◯ Divers
- ◯ Keine Angabe

Derzeit wohne ich in...

- ◯ Deutschland
- ◯ Österreich
- ◯ Schweiz
- ◯ Sonstiges (bitte angeben)

Meine vorrangige Sportart ist

- ◯ Laufen
- ◯ Radfahren
- ◯ Triathlon

Ich sehe mich selbst (in der Sportart Laufen, Triathlon oder Radfahren) als

- ◯ Hobbysportler (keine Teilnahme an Wettkämpfen oder maximal an regionalen Rennen/Wettkämpfen)
- ◯ Amateur-Sportler (Teilnahme an nationalen und/oder internationalen Rennen/Wettkämpfen)
- ◯ Profi-Sportler

UNIVERSITY of STIRLING SPORT

Warum trainierst Du?

Bitte wähle die zutreffendste Antwort aus

- ◯ Leistungsverbesserung
- ◯ Allgemeine Fitness und Gesundheit
- ◯ Sonstiges (bitte angeben)

UNIVERSITY of STIRLING SPORT

Regelmäßiges Ausdauertraining von mindestens 2 Stunden pro Woche, mache ich seit

- < 1 Jahr
- 1-3 Jahre
- 4-6 Jahre
- 7-10 Jahre
- > 10 Jahre

In den letzten 12 Monaten trainierte ich durchschnittlich (einschließlich aller Trainingsaktivitäten wie z.B. Krafttraining usw.)

- < 5 Stunden/Woche
- 5-9 Stunden/Woche
- 10-14 Stunden/Woche
- 15-20 Stunden/Woche
- 21-25 Stunden/Woche
- > 25 Stunden/Woche

Wenn ich Kohlenhydrate während dem Training (dazu zählt z.B. auch die Wettkampfvorbereitung) konsumiere, verwende ich normalerweise

Bitte wähle die zutreffendste Antwort aus (bezogen auf die letzten 12 Monate)

- Ausschließlich alltägliche Snacks/Getränke (z.B. Müsliriegel, Banane, Brötchen, Saftschorle, selbstgemachte Snacks/Getränke...)
- Ausschließlich kommerziell erhältliche Sporternährungsprodukte (z.B. Riegel, Gels, isotonische Sportgetränke von Sporternährungsherstellern)
- Unterschiedlich: entweder kommerziell erhältliche Sporternährungsprodukte (z.B. Riegel, Gels, isotonische Sportgetränke von Sporternährungsherstellern) oder alltägliche Snacks/Getränke (z.B. Müsliriegel, Banane, Brötchen, Apfelschorle, selbstgemachte Snacks/Getränke) oder beides in Kombination

Ich verwende normalerweise ausschließlich kommerziell erhältliche Sporternährungsprodukte (und keine alltäglichen Snacks/Getränke) für die Kohlenhydratzufuhr während des Trainings, weil:

Bitte wähle alle zutreffenden Gründe aus (bezogen auf die letzten 12 Monate)

- ☐ Zeitersparnis – keine Zubereitung (die Produkte sind bereits vorportioniert verpackt) oder nur einfache Zubereitung notwendig
- ☐ Einfache Vorratshaltung (lange Haltbarkeit der Produkte, keine Kühlung notwendig)
- ☐ Einfach zu konsumieren während der sportlichen Belastung
- ☐ Gute Transportierbarkeit (z.B. handlich, platzsparend, leicht)
- ☐ Ideales Nährwertprofil (z.B. hoher Kohlenhydratanteil und gleichzeitig niedriger Fettgehalt)
- ☐ Optimale Kohlenhydratzusammensetzung (z.B. Mix aus Fruktose- und Glukose, schnelle oder nur langsame Kohlenhydratarten)
- ☐ Optimaler Kohlenhydratgehalt pro Portionsgröße (z.B. pro Gel, Riegel, 500ml Getränk)
- ☐ Gute Verträglichkeit (keine Magen-Darmbeschwerden)
- ☐ Aus sensorischen Gründen (aufgrund von Geschmack, Geruch oder Textur der Produkte)
- ☐ Bessere sportliche Leistung möglich
- ☐ Aus Sponsoring-Gründen oder weil ich die Produkte kostenlos bekomme
- ☐ Gutes Preis-Leistungs-Verhältnis
- ☐ Meine Freunde, Teamkollegen, Konkurrenten oder Profisportler diese verwenden
- ☐ Mein Ernährungsberater, Trainer oder Coach es mir empfiehlt
- ☐ Sonstiges (bitte angeben)

UNIVERSITY of STIRLING SPORT

Ich verwende normalerweise ausschließlich alltägliche Snacks/Getränke (und keine kommerziell erhältlichen Sporternährungsprodukte) für die Kohlenhydratzufuhr während des Trainings, weil:

Bitte wähle alle zutreffenden Gründe aus (bezogen auf die letzten 12 Monate)

- ☐ Gesunde Lebensmittel und natürliche Zutaten sind mir wichtig
- ☐ Ich gerne Sporternährungssnacks/-drinks selbst zubereite bzw. selbstgemachte Snacks/Getränke verwende
- ☐ Aus sensorischen Gründen (aufgrund von Geschmack, Geruch oder Textur der Lebensmittel)
- ☐ Gute Verträglichkeit (keine Magen-Darmbeschwerden)
- ☐ Kommerziell erhältliche Sporternährungsprodukte sind mir zu künstlich bzw. zu wenig natürlich
- ☐ „Preis“, kommerziell erhältliche Sporternährungsprodukte sind mir zu teuer
- ☐ Kommerziell erhältliche Sporternährungsprodukte habe ich bisher noch nie ausprobiert
- ☐ Ich der Meinung bin, dass ich keine Sporternährungsprodukte brauche
- ☐ Aus Gewohnheit
- ☐ Meine Freunde, Teamkollegen, Konkurrenten oder Profisportler das so machen
- ☐ Mein Ernährungsberater, Trainer oder Coach es mir empfiehlt
- ☐ Gutes Preis-Leistungs-Verhältnis
- ☐ Anti-Doping: Bei alltäglichen Lebensmitteln bin ich mir sicher, dass sie frei von verbotenen Substanzen im Sport sind
- ☐ Sonstiges (bitte angeben)

UNIVERSITY of STIRLING SPORT

Ich verwende entweder kommerziell erhältliche Sporternährungsprodukte oder alltägliche Snacks/Getränke (oder auch beides in Kombination) für die Kohlenhydratzufuhr während des Trainings, weil:

Bitte wähle alle zutreffenden Gründe aus (bezogen auf die letzten 12 Monate)

- ☐ Grundsätzlich bevorzuge ich alltägliche Lebensmittel. In manchen Trainingssituationen (z.B. bei hohen Intensitäten, langen Einheiten oder Wettkampfvorbereitung) sind jedoch Sporternährungsprodukte praktischer
- ☐ Manchmal ist es einfacher/zeitsparender auf Sporternährungsprodukte zurückzugreifen, da lediglich eine einfache Zubereitung oder gar keine Zubereitung (Produkte sind bereits vorportioniert abgepackt) notwendig ist, ansonsten nutze ich alltägliche Lebensmittel
- ☐ Je nach Verfügbarkeit in meiner Nähe (zu Hause, auf Reisen etc.), verwende ich entweder Sporternährungsprodukte oder alltägliche Lebensmittel
- ☐ Aus Verträglichkeitsgründen: Eine Kombination aus Sporternährungsprodukten und alltäglichen Lebensmitteln vertrage ich bei langen Trainingseinheiten am besten
- ☐ Aus Verträglichkeitsgründen nutze ich Sporternährungsprodukte vor allem bei hohen Trainingsintensitäten, ansonsten gerne auch alltägliche Lebensmittel
- ☐ Neben alltäglichen Lebensmitteln nutze ich auch Sporternährungsprodukte, da diese für mich kostenfrei sind (z.B. Sponsoring)
- ☐ Grundsätzlich bevorzuge ich Sporternährungsprodukte, jedoch kann oder will ich mir diese Produkte nicht immer leisten
- ☐ Damit ich „sensorische“ (Geschmack, Textur oder Geruch von alltäglichen Lebensmitteln und Sporternährungsprodukten) Abwechslung habe
- ☐ Meine Freunde, Teamkollegen, Konkurrenten oder Profisportler das so machen
- ☐ Mein Ernährungsberater, Trainer oder Coach es mir empfiehlt
- ☐ Sonstiges (bitte angeben)

UNIVERSITY of STIRLING SPORT

Wenn ich Kohlenhydrate während dem Training konsumiere, verwende ich bei <u>niedrigen bis mittleren Belastungsintensitäten</u>*

**Klassifizierung der Belastungsintensität: Falls nicht anderweitig gesteuert, nach subjektivem Belastungsempfinden: „leicht" bis "etwas anstrengend“.*

	(in keiner Trainingseinheit, bei denen ich Kohlenhydrate zu mir nehme) **Nie**	(in max. 10% der Trainingseinheiten, bei denen ich Kohlenhydrate zu mir nehme) **Selten**	*(in bis zu 50% der Trainingseinheiten, bei denen ich Kohlenhydrate zu mir nehme)* **Manchmal**	*(mehr als 50% der Trainingseinheiten, bei denen ich Kohlenhydrate zu mir nehme)* **Häufig**	*(in allen Trainingseinheiten, bei denen ich Kohlenhydrate zu mir nehme)* **Immer**
**Sportgetränke (z.B. isotonische Sports Drinks)**	○	○	○	○	○
**Gels**	○	○	○	○	○
**Energie/Kohlenhydratriegel**	○	○	○	○	○
**Sporternährungs-Süßigkeiten (z.B. Gummibonbons, Energy chews)**	○	○	○	○	○

Sonstiges (bitte zusätzlich angeben ob: "Selten", "Manchmal", "Häufig", "Immer"

Wenn ich Kohlenhydrate während dem Training konsumiere, verwende ich bei <u>hohen Belastungsintensitäten</u>*

**Klassifizierung der Belastungsintensität: Falls nicht anderweitig gesteuert, nach subjektivem Belastungsempfinden: „anstrengend" bis "extrem anstrengend“.*

	(in keiner Trainingseinheit, bei denen ich Kohlenhydrate zu mir nehme) **Nie**	*(in max. 10% der Trainingseinheiten, bei denen ich Kohlenhydrate zu mir nehme)* **Selten**	*(in bis zu 50% der Trainingseinheiten, bei denen ich Kohlenhydrate zu mir nehme)* **Manchmal**	*(mehr als 50% der Trainingseinheiten, bei denen ich Kohlenhydrate zu mir nehme)* **Häufig**	*(in allen Trainingseinheiten, bei denen ich Kohlenhydrate zu mir nehme)* **Immer**
**Sportgetränke (z.B. isotonische Sports Drinks)**	○	○	○	○	○
**Gels**	○	○	○	○	○
**Energie/Kohlenhydratriegel**	○	○	○	○	○
**Sporternährungs-Süßigkeiten (z.B. Gummibonbons, Energy chews)**	○	○	○	○	○

Sonstiges (bitte zusätzlich angeben ob: "Selten" "Manchmal", "Häufig", "Immer"):

Wenn ich Kohlenhydrate während dem Training konsumiere, verwende ich bei niedrigen bis mittleren Belastungsintensitäten*

**Klassifizierung der Belastungsintensität: Falls nicht anderweitig gesteuert, nach subjektivem Belastungsempfinden: „leicht" bis "etwas anstrengend“.*

	(in keiner Trainingseinheit, bei denen ich Kohlenhydrate zu mir nehme) **Nie**	*(in max. 10% der Trainingseinheiten, bei denen ich Kohlenhydrate zu mir nehme)* **Selten**	*(in bis zu 50% der Trainingseinheiten, bei denen ich Kohlenhydrate zu mir nehme)* **Manchmal**	*(mehr als 50% der Trainingseinheiten, bei denen ich Kohlenhydrate zu mir nehme)* **Häufig**	*(in allen Trainingseinheiten, bei denen ich Kohlenhydrate zu mir nehme)* **Immer**
**Cola, Energy Drinks (z.B. Red Bull)**	○	○	○	○	○
**Gekaufte, gesüßte Getränke (z.B. Ice Tea, Limonade), Saftschorlen**	○	○	○	○	○
**Selbstgemachte Sportgetränke**	○	○	○	○	○
**Trockenobst (z.B. Rosinen, Datteln...)**	○	○	○	○	○
**Banane**	○	○	○	○	○
**Brot/Brötchen/Laugengebäck pur oder mit Aufstrich**	○	○	○	○	○
**Sandwiches/belegte Brote**	○	○	○	○	○
**Selbstgemachte süße Snacks (z.B. Reiskuchen, Müsliriegel, Kuchen/Kekse...)**	○	○	○	○	○
**Gekaufte Müsli-, Frucht- oder andere Energieriegel**	○	○	○	○	○
**Gekaufte, süße Backwaren (z.B. Kuchen, Muffins oder Kekse...)**	○	○	○	○	○
**Quetschbeutel (Fruchtpürees, z.B. von Hipp)**	○	○	○	○	○
**Süßigkeiten (z.B. Gummibärchen, Schokolade)**	○	○	○	○	○

Sonstiges (bitte zusätzlich angeben ob: "Selten" "Manchmal", "Häufig", "Immer"):

Wenn ich Kohlenhydrate während dem Training konsumiere, verwende ich bei <u>hohen Belastungsintensitäten</u>*

**Klassifizierung der Belastungsintensität: Falls nicht anderweitig gesteuert, nach subjektivem Belastungsempfinden: „anstrengend" bis "extrem anstrengend“.*

	(in keiner Trainingseinheit, bei denen ich Kohlenhydrate zu mir nehme) **Nie**	*(in max. 10% der Trainingseinheiten, bei denen ich Kohlenhydrate zu mir nehme)* **Selten**	*(in bis zu 50% der Trainingseinheiten, bei denen ich Kohlenhydrate zu mir nehme)* **Manchmal**	*(mehr als 50% der Trainingseinheiten, bei denen ich Kohlenhydrate zu mir nehme)* **Häufig**	*(in allen Trainingseinheiten, bei denen ich Kohlenhydrate zu mir nehme)* **Immer**
**Cola, Energy Drinks (z.B. Red Bull)**	○	○	○	○	○
**Gekaufte, gesüßte Getränke (z.B. Ice Tea, Limonade), Saftschorlen**	○	○	○	○	○
**Selbstgemachte Sportgetränke**	○	○	○	○	○
**Trockenobst (z.B. Rosinen, Datteln...)**	○	○	○	○	○
**Banane**	○	○	○	○	○
**Brot/Brötchen/Laugengebäck pur oder mit Aufstrich**	○	○	○	○	○
**Sandwiches/belegte Brote**	○	○	○	○	○
**Selbstgemachte süße Snacks (z.B. Reiskuchen, Müsliriegel, Kuchen/Kekse...)**	○	○	○	○	○
**Gekaufte Müsli-, Frucht- oder andere Energieriegel**	○	○	○	○	○
**Gekaufte, süße Backwaren (z.B. Kuchen, Muffins oder Kekse...)**	○	○	○	○	○
**Quetschbeutel (Fruchtpürees, z.B. von Hipp)**	○	○	○	○	○
**Süßigkeiten (z.B. Gummibärchen, Schokolade)**	○	○	○	○	○

Sonstiges (bitte zusätzlich angeben ob: "Selten" "Manchmal", "Häufig", "Immer"):

[ ]

Wenn ich Kohlenhydrate während dem Training konsumiere, verwende ich bei <u>niedrigen bis mittleren Belastungsintensitäten</u>*

**Klassifizierung der Belastungsintensität: Falls nicht anderweitig gesteuert, nach subjektivem Belastungsempfinden: „leicht" bis "etwas anstrengend".*

a) folgende kommerziell erhältliche Sporternährungsprodukte

	(in keiner Trainingseinheit, bei denen ich Kohlenhydrate zu mir nehme) **Nie**	*(in max. 10% der Trainingseinheiten, bei denen ich Kohlenhydrate zu mir nehme)* **Selten**	*(in bis zu 50% der Trainingseinheiten, bei denen ich Kohlenhydrate zu mir nehme)* **Manchmal**	*(mehr als 50% der Trainingseinheiten, bei denen ich Kohlenhydrate zu mir nehme)* **Häufig**	*(in allen Trainingseinheiten, bei denen ich Kohlenhydrate zu mir nehme)* **Immer**
**Sportgetränke (z.B. isotonische Sports Drinks)**	○	○	○	○	○
**Gels**	○	○	○	○	○
**Energie/Kohlenhydratriegel**	○	○	○	○	○
**Sporternährungs-Süßigkeiten (z.B. Gummibonbons, Energy chews)**	○	○	○	○	○

Sonstiges (bitte zusätzlich angeben ob: "Selten" "Manchmal", "Häufig", "Immer"):

[ ]

Wenn ich Kohlenhydrate während dem Training konsumiere, verwende ich bei <u>niedrigen bis mittleren Belastungsintensitäten</u>*

**Klassifizierung der Belastungsintensität: Falls nicht anderweitig gesteuert, nach subjektivem Belastungsempfinden: „leicht" bis "etwas anstrengend“.*

b) folgende alltägliche Snacks/Getränke

	(in keiner Trainingseinheit, bei denen ich Kohlenhydrate zu mir nehme) **Nie**	*(in max. 10% der Trainingseinheiten, bei denen ich Kohlenhydrate zu mir nehme)* **Selten**	*(in bis zu 50% der Trainingseinheiten, bei denen ich Kohlenhydrate zu mir nehme)* **Manchmal**	*(mehr als 50% der Trainingseinheiten, bei denen ich Kohlenhydrate zu mir nehme)* **Häufig**	*(in allen Trainingseinheiten, bei denen ich Kohlenhydrate zu mir nehme)* **Immer**
**Cola, Energy Drinks (z.B. Red Bull)**	○	○	○	○	○
**Gekaufte, gesüßte Getränke (z.B. Ice Tea, Limonade), Saftschorlen**	○	○	○	○	○
**Selbstgemachte Sportgetränke**	○	○	○	○	○
**Trockenobst (z.B. Rosinen, Datteln...)**	○	○	○	○	○
**Banane**	○	○	○	○	○
**Brot/Brötchen/Laugengebäck pur oder mit Aufstrich**	○	○	○	○	○
**Sandwiches/belegte Brote**	○	○	○	○	○
**Selbstgemachte süße Snacks (z.B. Reiskuchen, Müsliriegel, Kuchen/Kekse...)**	○	○	○	○	○
**Gekaufte Müsli-, Frucht- oder andere Energieriegel**	○	○	○	○	○
**Gekaufte, süße Backwaren (z.B. Kuchen, Muffins oder Kekse...)**	○	○	○	○	○
**Quetschbeutel (Fruchtpürees, z.B. von Hipp)**	○	○	○	○	○
**Süßigkeiten (z.B. Gummibärchen, Schokolade)**	○	○	○	○	○

Sonstiges (bitte zusätzlich angeben ob: "Selten", "Manchmal", "Häufig", "Immer")

____

Wenn ich Kohlenhydrate während dem Training konsumiere, verwende ich bei <u>hohen Belastungsintensitäten</u>*

**Klassifizierung der Belastungsintensität: Falls nicht anderweitig gesteuert, nach subjektivem Belastungsempfinden: „anstrengend" bis "extrem anstrengend“.*

a) folgende kommerziell erhältliche Sporternährungsprodukte

	(in keiner Trainingseinheit, bei denen ich Kohlenhydrate zu mir nehme) ***Nie***	*(in max. 10% der Trainingseinheiten, bei denen ich Kohlenhydrate zu mir nehme)* **Selten**	*(in bis zu 50% der Trainingseinheiten, bei denen ich Kohlenhydrate zu mir nehme)* **Manchmal**	*(mehr als 50% der Trainingseinheiten, bei denen ich Kohlenhydrate zu mir nehme)* **Häufig**	*(in allen Trainingseinheiten, bei denen ich Kohlenhydrate zu mir nehme)* **Immer**
**Sportgetränke (z.B. isotonische Sports Drinks)**	○	○	○	○	○
**Gels**	○	○	○	○	○
**Energie/Kohlenhydratriegel**	○	○	○	○	○
**Sporternährungs-Süßigkeiten (z.B. Gummibonbons, Energy chews)**	○	○	○	○	○

Sonstiges (bitte zusätzlich angeben ob: "Selten" "Manchmal", "Häufig", "Immer"):

[ ]

Wenn ich Kohlenhydrate während dem Training konsumiere, verwende ich bei <u>hohen Belastungsintensitäten</u>*

**Klassifizierung der Belastungsintensität: Falls nicht anderweitig gesteuert, nach subjektivem Belastungsempfinden: „anstrengend" bis "extrem anstrengend“.*

b) folgende alltägliche Snacks/Getränke

	(in keiner Trainingseinheit, bei denen ich Kohlenhydrate zu mir nehme) **Nie**	*(in max. 10% der Trainingseinheiten, bei denen ich Kohlenhydrate zu mir nehme)* **Selten**	*(in bis zu 50% der Trainingseinheiten, bei denen ich Kohlenhydrate zu mir nehme)* **Manchmal**	*(mehr als 50% der Trainingseinheiten, bei denen ich Kohlenhydrate zu mir nehme)* **Häufig**	*(in allen Trainingseinheiten, bei denen ich Kohlenhydrate zu mir nehme)* **Immer**
**Cola, Energy Drinks (z.B. Red Bull)**	○	○	○	○	○
**Gekaufte, gesüßte Getränke (z.B. Ice Tea, Limonade), Saftschorlen**	○	○	○	○	○
**Selbstgemachte Sportgetränke**	○	○	○	○	○
**Trockenobst (z.B. Rosinen, Datteln...)**	○	○	○	○	○
**Banane**	○	○	○	○	○
**Brot/Brötchen/Laugengebäck pur oder mit Aufstrich**	○	○	○	○	○
**Sandwiches/belegte Brote**	○	○	○	○	○
**Selbstgemachte süße Snacks (z.B. Reiskuchen, Müsliriegel, Kuchen/Kekse...)**	○	○	○	○	○
**Gekaufte Müsli-, Frucht- oder andere Energieriegel**	○	○	○	○	○
**Gekaufte, süße Backwaren (z.B. Kuchen, Muffins oder Kekse...)**	○	○	○	○	○
**Quetschbeutel (Fruchtpürees, z.B. von Hipp)**	○	○	○	○	○
**Süßigkeiten (z.B. Gummibärchen, Schokolade)**	○	○	○	○	○

Sonstiges (bitte zusätzlich angeben ob: "Selten", "Manchmal", "Häufig", "Immer")

[ ]

UNIVERSITY of STIRLING SPORT

Ich richte meinen Kohlenhydratbedarf bzw. die benötigte Kohlenhydratmenge im Training nach

**Bitte wähle die zutreffendste Antwort aus*

- ◯ Bauchgefühl
- ◯ Gramm Kohlenhydrate pro Stunde
- ◯ Empfehlung meines Coaches oder Ernährungsberaters
- ◯ Glukose Biosensor (z.B. Supersapiens)
- ◯ Sonstiges (bitte angeben)

UNIVERSITY of STIRLING SPORT

Abhängig von Trainingseinheit und Zielsetzung, nehme ich bis zu folgende Kohlenhydratmengen pro Stunde zu mir...

**Bitte wähle die zutreffendste Antwort aus*

- ◯ bis zu 25g Kohlenhydrate pro Stunde
- ◯ bis zu 26-30g Kohlenhydrate pro Stunde
- ◯ bis zu 31-40g Kohlenhydrate pro Stunde
- ◯ bis zu 41-50g Kohlenhydrate pro Stunde
- ◯ bis zu 51-60g Kohlenhydrate pro Stunde
- ◯ bis zu 61-70g Kohlenhydrate pro Stunde
- ◯ bis zu 71-80g Kohlenhydrate pro Stunde
- ◯ bis zu 81-90g Kohlenhydrate pro Stunde
- ◯ bis zu 91-100g Kohlenhydrate pro Stunde
- ◯ bis zu 101-125g Kohlenhydrate pro Stunde
